# Liensinine Inhibits Cell Growth and Blocks Autophagic Flux in Nonsmall-Cell Lung Cancer

**DOI:** 10.1155/2022/1533779

**Published:** 2022-07-01

**Authors:** Minghui Chang, Shanshan Ding, Xiaohan Dong, Xiaoling Shang, Yang Li, Li Xie, Xingguo Song, Xianrang Song

**Affiliations:** ^1^Department of Clinical Laboratory, Shandong Cancer Hospital and Institute, Shandong First Medical University and Shandong Academy of Medical Sciences, Jinan, Shandong, China; ^2^Shandong Provincial Key Laboratory of Radiation Oncology, Shandong Cancer Hospital and Institute, Shandong First Medical University and Shandong Academy of Medical Sciences, Shandong, China

## Abstract

Liensinine is a bioactive component of Plumula Nelumbinis extracted from the green embryo of the mature seeds of Nelumbonaceae and exhibits therapeutic functions and noteworthy anti-tumor effects in recent studies. However, the potential anti-tumor property and the underlying mechanisms of liensinine in nonsmall-cell lung cancer (NSCLC) have not been illustrated. In this study, we demonstrated that liensinine has the potential anti-tumor property, and it could inhibit growth of NSCLC *in vitro* and *in vivo*. In addition, we found that although it induced significant accumulation of autophagosomes, liensinine could quench them for degradation and blocked autophagic flux. Importantly, we observed that liensinine inhibited the normal function of mitochondrial energy supply and impaired the lysosomal function. This research firstly provides a possibility insight that liensinine could be a novel therapeutic strategy for NSCLC.

## 1. Introduction

Traditional Chinese medicines have been widely used by Chinese doctors in the treatment of intractable diseases for thousands of years. Recently, they attract attention from Western clinicians and researchers, due to the fact traditional Chinese medicines have wide applications and lower side effects [1, 2]. An increasing number of practices have proved that ingredients in Chinese herbal medicine could be used to deal with various cancers and are considered a great treasure house for searching for novel therapeutic agents [3, 4].

Liensinine is an active component extracted from the green embryo of the mature seeds of Nelumbonaceae [5]. Previous studies reported that liensinine could antagonize the ventricular arrhythmias [6], relax smooth muscle, and also have a hypotensive effect and a direct vasodilative effect [1]. This nature compound recently attracts great attention due to accumulating data that demonstrates its anti-tumor effects in several types of malignancy, such as gastric cancer [7], gallbladder cancer [8], colorectal cancer [9], and breast cancer [10, 11]. It has also been reported that liensinine sensitizes chemotherapy through DNM1L-mediated mitochondrial fission in breast cancer cells [11]. Another study found that liensinine induces gallbladder cancer cell apoptosis and G2/M arrest by impacting the PI3K/AKT signaling pathway [8]. However, the anti-tumor effects and the underlying mechanisms of liensinine in NSCLC have not been illustrated.

NSCLC is considered the leading type of cancers worldwide and accounts for approximately eighty percent of lung cancer cases [12]. While the pace of the annual decline in overall lung cancer mortality doubled from 2.4% to 5% during 2014 to 2018 [13], it still causes the most deaths, far exceeding any other types of all cancers [13]. Despite standard guidelines and management for NSCLC therapy [14, 15], the exploration and development of novel therapeutic approaches and more effective agents are meaningful to minimize the dreadful burden caused by this disease.

Mitochondrion is a critical component of the cell that regulates the generation of cellular energy by oxidative phosphorylation as well as the execution of cell apoptosis, death, and intracellular redox homeostasis [16, 17]. It is no doubt that mitochondria are functional in cancer cells [18, 19]. Mainstream research demonstrated that cancer cells generate adenosine triphosphate for their survival mostly based on aerobic glycolysis. Cristina et al. reported that the *β*1-blocker nebivolol specifically inhibits mitochondria complex and adenosine triphosphate (ATP) synthase activities, causing a metabolic transformation and oxidative stress crisis, thus slowing down the growth of cancer cells [20]. Another study reported that lonidamine induces autophagic cell death and inhibits the proliferation and metastasis of tumors by restricting the mitochondrial bioenergetics via inactivating the AKT/mTOR/p70S6K signaling pathway [21]. Therefore, seeking novel agents to target mitochondrial bioenergetics might be a feasible approach to defeat cancer cells.

In this study, we performed a series of assays to clarify the anti-tumor effect of liensinine in NSCLC cells. We found that liensinine inhibits the growth of NSCLC cells *in vitro* by inducing apoptosis, which was also confirmed *in vivo*. Liensinine also causes mitochondrial damage in NSCLC cells compared to the control groups. More importantly, we observed that liensinine functions as an autophagy inhibitor in NSCLC by blocking the autophagy at a late stage. This is possibly because liensinine inhibits the normal function of mitochondrial energy supply and impairs the lysosomal function. Therefore, our research is the first to suggest that liensinine can be a novel agent for NSCLC therapy.

## 2. Materials and Methods

### 2.1. Cell Lines and Compounds

A549, SPC-A1, and NCI–H520, three human nonsmall-cell lung cancer cell lines were purchased from China Center for Type Culture Collection (Wuhan, China). All these cells were cultured in DMEM (11965084, Gibco/Invitrogen, USA) supplemented with 10% fetal bovine serum (10100139C, Gibco/Invitrogen, USA) and penicillin/streptomycin (C0222, Beyotime, China) at 37°C in 5% CO^2^. Liensinine was purchased from MedChemExpress (HY–N0484, China), bafilomycin A1 was purchased from Sigma-Aldrich (Shanghai, China), Z-VAD-FMK was purchased from Keygen Biotech (Jiangsu, China), and all these compounds were dissolved in DMSO (D2650, Sigma-Aldrich, China) and stored at −20 °C. Antibodies include the following: cleaved-PARP (#5625), caspase 3 (#2723), cleaved-caspase 3 (#9661), cleaved-caspase 9 (#9509), BAX (#5023), cytochrome *c* (#11940), LC3B (#3868), SQSTM1 (#5114), mTOR (#2983), phospho-mTOR (#5536), AMPK (#5831), phospho-AMPK (#50081), Beclin1 (#3495), ULK1 (#8054), and anti-rabbit IgG (#7074) were obtained from Cell Signaling Technology (Danvers, USA). LAMP2 (No. 66301-1-Ig) and ACTB (No. 20536-1-AP) were obtained from Proteintech (Wuhan, China). LAMP1 (sc-20011) was obtained from Santa Cruz Biotechnology (Dallas, USA).

### 2.2. CCK-8 Assay

CCK-8 assays were performed to detect the cytotoxic effects of liensinine according to the description provided by the manufacturer (C0037, Beyotime, China). Three cell lines were seeded into 96-well plates with 3000 cells every well and were incubated for 24h. Liensinine was prepared for different concentrations (0, 10, 20, 40, 60, and 80 *μ*M) and added into the wells, incubating for 24 h or 48h. After incubation for different times, CCK-8 solution was added and incubated for 2 h. SpectraMax i3 (Molecular Devices, USA) was used to measure the absorbance at 450 nm.

### 2.3. Colony Formation Assay

The colony formation assays were performed to evaluate the growth ability of cells under liensinine treatment. Three cell lines were seeded into 6-well plates with 300–500 cells each well. Liensinine was prepared for different concentrations (0, 2.5, 5, 10, or 20 *μ*M) and added into the wells for 48 h, and the culture medium with liensinine was taken out and incubated with normal culture medium for 10–14 days. When the colony exceeded 50 cells, the cells were fixed with acetic acid-methanol (1 : 4) and stained with diluted crystal violet (1 : 30). And then, the colonies were counted and calculated. The colony formation efficiency was calculated with the following formula: survival fraction = clones/cell numbers × 100%.

### 2.4. Apoptosis Analysis

Annexin V-FITC Apoptosis Detection Kit (556547, BD Biosciences Pharmingen, USA) was used to detect the apoptotic cells following the manufacturer's instructions. Three cell lines were seeded into 6-well plates with 1^*∗*^10^5^ cells each well. Liensinine was prepared for different concentrations (0, 2.5, 5, 10, or 20 *μ*M) and added into the wells for 48h. After that, the cells were stained with FITC annexin V and PI for 15–30 minutes in the dark, then FACS Calibur instrument (Becton, Dickinson and Company, Bedford, MA, USA) was used to detect the fluorescence signal, and the stained cell population were presented. Data were analyzed using FlowJo software 7.6.

### 2.5. Western Blot

Cells were seeded into 6-well plates with 1^*∗*^10^5^ cells each well. Liensinine was prepared for different concentrations (0, 2.5, 5, 10, or 20 *μ*M) and added into the wells for 48h. After washing PBS for 3 times, cells were lysed with lysis buffer (P0013 J, Beyotime, China) supplemented with protease inhibitor (P1005, Beyotime, China) and phosphatase inhibitor (P1081, Beyotime, China). After centrifuging at 12000 × *g* for 15 min at 4°C, the concentration of the supernatant was detected by a BCA kit (23225, Thermo Fisher Scientific, USA). And then, Western blot assay was carried out in a routine process [22]. The results were visualized using ECL substrate reagent kit (32209, Thermo Fisher Scientific, USA) or detected by exposure to a film. The primary antibody dilution is as follows: cleaved-PARP (1 : 1000), caspase 3 (1 : 1000), cleaved-caspase 3 (1 : 500), cleaved-caspase 9 (1 : 500), BAX (1 : 1000), cytochrome *c* (1 : 1000), LC3B (1 : 500), SQSTM1 (1 : 1000), mTOR (1 : 1000), phospho-mTOR (1 : 1000), AMPK (1 : 1000), phospho-AMPK (1 : 1000), Beclin1 (1 : 1000), ULK1 (1 : 1000), LAMP2 (1 : 1000), LAMP1 (1 : 1000), and ACTB (1 : 1000). Anti-rabbit IgG was diluted at a ratio of 1 : 2000. Primary antibody was incubated overnight at 4°C, and secondary antibody was incubated for 1 hour.

### 2.6. *In vivo* Studies

Four- to six-week-old female BALB/*c* nude mice were purchased from Beijing Huafukang Bioscience Co. Ltd. (Beijing, China). Mice were housed and handled in laminar flow cabinets under specific pathogen-free conditions with temperature at 25 °C ± 2°C and a relative humidity of 70% ± 5% according to institutional guidelines and experimental procedures approved by the Institutional Animal Care and Use Committee of Shandong *Cancer* Hospital affiliated Shandong First Medical University. For constructing the model, approximately 5^*∗*^10^6^ A549 cells suspending in 100 *μ*l PBS were injected into the right flank of nude mice. When most tumors reached 100 mm^3^ in size, we randomly divided these mice into three groups with six per group. Three groups of mice were injected intraperitoneally with DMSO, liensinine (5 mg/kg), or liensinine (20 mg/kg) every two days. The volume of local tumors was observed and calculated by measuring two perpendicular diameters (length and width) every two days using a caliper. The mouse body weight was calculated every two days using an electronic scale. The volume was calculated following the formula: tumor volume (mm^3^) = 1/2  × (length × square width). On the 25th day, mice were sacrificed according to the 2020 AVMA Guidelines on Euthanasia state. Mice were injected intraperitoneally with 0.1 ml 1% sodium phenobarbital for anesthesia, and then the spinal cord was disconnected from the brain with force and speed. After that, tumors were dissected and weighted. Their hearts, livers, spleens, lungs, and kidneys were also dissected, and HE and IHC assays were performed.

### 2.7. H&E and IHC Staining

After the mice were sacrificed, tumors, hearts, livers, spleens, lungs, and kidneys were resected and immediately fixed in 10% formalin. The slides were deparaffinized and stained with hematoxylin, rinsed with distilled water, rinsed with 0.1% hydrochloric acid in 50% ethanol, rinsed with tap water, stained with eosin, and rinsed again with distilled water. The slides were dehydrated and mounted with coverslips. After routinely dewaxing and hydration, antigen in specimens proceeded to be repaired, and the slides were blocked for endogenous peroxidase activity, preincubated with goat serum, and then stained with cleaved-caspase 3 antibody (GB11532, Servicebio Technology, China). Secondary staining was carried out with HRP-conjugated anti-rabbit IgG and DAB peroxidase substrate.

### 2.8. JC-1 Assay

JC-1 Assay Kit (C2006, Beyotime, China) was used to detect the mitochondrial membrane potential following the manufacturer's instructions. Three cell lines were seeded into 6-well plates with 1^*∗*^10^5^ cells each well. Liensinine was prepared for different concentrations (0, 2.5, 5, 10, or 20 *μ*M) and added into the wells for 48 h. After liensinine treatment and incubation, the cells were stained with JC-1 reagent for 20 min in the dark. FACS Calibur instrument (Becton Dickinson, USA) was used to detect the fluorescence signal, and the stained cell population were presented. Data were analyzed using FlowJo software 7.6.

### 2.9. ATP Assay

ATP level was detected using ATP Assay Kit (S0026, Beyotime, China) following the manufacturer's instructions. Cells were dissociated in the ice and centrifuged at 12000 × g for 5 min at 4°C to take the supernatant, and then we added diluted ATP detection reagent. After mixing thoroughly, the samples measured the absorbance immediately using SpectraMax i3 (Molecular Devices, USA).

### 2.10. Immunofluorescence

Cells were seeded into 12-well plates with 5^*∗*^10^5^ cells each well. After cells were treated, 4% paraformaldehyde and 0.1% Triton X-100 were added to fix the cells for 10–15 min. And then, cells were washed with PBS for three times blocking more than 0.5 h. The primary antibodies were added and incubated overnight, and cells were incubated for 1 h with secondary antibodies. Finally, DAPI was added and stained the nuclei of cells for 3 min. The primary antibodies are as follows: LC3B (#3868), SQSTM1 (#88588), and COX IV (#4850) were purchased from Cell Signaling Technology (Danvers, USA). The secondary antibodies are as follows: Alexa Fluor 488 IgG (A0423) and Alexa Fluor 555 IgG (A0460) were purchased from Beyotime (Shanghai, China). Confocal laser microscopy (LSM800, Carl Zeiss, Germany) was used to observe and acquire the pictures.

### 2.11. Adenovirus Infection and Cell Transfection

Recombinant adenovirus-mRFP-GFP-LC3 was purchased from HanBio (Wuhan, China). A549 or SPC-A1 cells were seeded into 12-well plates with 5^*∗*^10^4^ cells each well and infected with adenovirus-mRFP-GFP-LC3 at a MOI of two for 24 h. Liensinine, Baf.A1, and EBSS were prepared for appropriate concentrations and added into the wells for 48 h. For transient gene silencing, a siRNA oligonucleotide targeting ATG5 was used. The ATG5 siRNA sequence was 5′-CCTTTCATTCAGAAGCTGTTT-3′. Lipofectamine 3000 (Invitrogen) was used for cell transfection following the manufacturer's instructions.

### 2.12. Transmission Electron Microscope

After treatment, TEM stationary solution (2.5% glutaraldehyde in 0.2 M HEPES) was used to fix the cells at 4°C for 4 h. The process of sample preparation was following the instructions as indicating previously [22]. The results were analyzed using TEM (HT7700, HITACHI), and Fiji ImageJ software was used to calculate and analyze the TEM pictures.

### 2.13. mtDNA Integrity Analysis

DNA lesions and content of mitochondrial genome were detected as methods described previously [23–28]. After drug treatment and incubation, the DNA of cells was isolated by using cell genomic DNA extraction kit (DP304, TIANGEN Biotech, China) according to the description by the manufacturer. Long-range PCR strategy was used to amplify an 8.9 kb region of mtDNA by using Takara LA Taq according to the description by the manufacturer. Real-Time PCR strategy was used to assess the 221 bp region of mtDNA and mtDNA content. All primers are listed in Table S1. The numbers of lesions/mitochondrial genome were calculated by using formula *L* = log (AT/A0)×1.12 (correction factor 1.12 = 10kb/8.9kb), where A is LR-PCR/mtDNA content (AT is the value for the treated sample, and A0 is the value for the control sample). PCR products were assessed by using agarose gel electrophoresis, and the results were calculated by Image*J*.

### 2.14. Targeted Metabolic

After drug incubation and treatment, cell metabolites were extracted with 80% methanol aqueous solution for LC-MS/MS analysis. Data acquisition system mainly includes ultra-performance liquid chromatography (UPLC) and tandem mass spectrometry (MS/MS). The data and contained information were qualitatively analyzed based on the self-established standard database (Lianchuan Biotech, Hangzhou). The Analyst 1.6.3 was used to process the mass spectrum data, and then principal component analysis and bioinformatics analysis of metabolic were performed.

### 2.15. Flow Cytometry for Fluorescence Probe Detection

Liensinine was prepared for different concentrations (0, 2.5, 5, 10, or 20 *μ*M) and added into the wells for 48 h. After liensinine treatment and incubation, cells were collected and loaded with Lyso-Tracker Red (LTR) (C1046, Beyotime, China) at 37°C for 30 min. After washing 3 times with PBS, the fluorescence intensities were measured by a flow cytometry.

### 2.16. Statistical Analysis

GraphPad Prism 6.02 (GraphPad Software, USA) was used for data analysis. Statistical analysis was carried out using Student's *t*-test for two groups as well as one-way ANOVA for more than two groups. Data are presented as the mean ± SD. For all statistical tests, significance was established at *P* < 0.05.

## 3. Results

### 3.1. Liensinine Exhibits Anti-Tumor Effects of NSCLC Cells *In Vitro*

**Figure 1:**
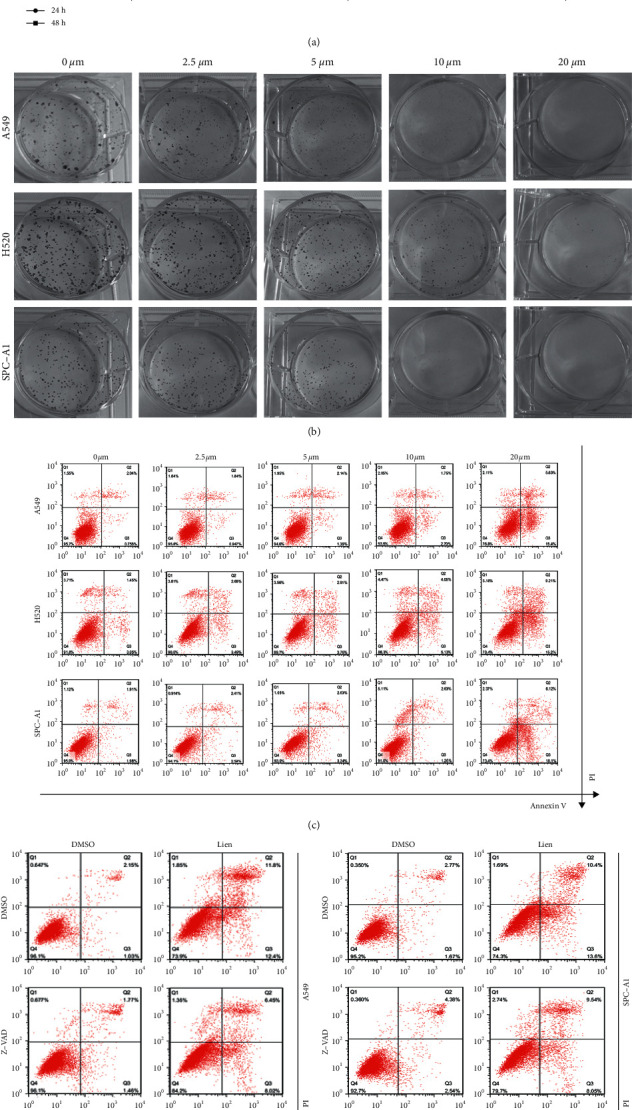
Liensinine exerts cytotoxicity and induces apoptosis in vitro. (a) A549, H520, and SPC-A1 cells were treated with the indicated concentrations of liensinine for 24h and 48h, respectively, and then CCK-8 assay was performed. (b) A549, H520, and SPC-A1 cells were treated with the indicated concentrations of liensinine for 48h and then incubated with normal culture for 14 days. (c) A549, H520, and SPC-A1 cells were treated with indicated concentrations of liensinine for 48h, and apoptosis assays were performed using annexin V and PI staining. (d) A549 and SPC-A1 cells were treated with 20 *μ*M liensinine with or without Z-VAD, and then apoptosis assay was performed. Three independent experiments were performed. The results were shown as means ± SD, ^*∗∗∗∗*^*P* < 0.0001.

To verify the cytotoxic effects of liensinine against NSCLC, A549, H520, and SPC-A1 cells were treated with DMSO or indicated concentrations of liensinine, and then subjected to CCK-8 assay. As shown in Figure 1(a), liensinine exhibited toxic effects in NSCLC cells in a concentration-dependent manner. In addition, colony formation assay was carried out to verify its effects. Liensinine also inhibited the numbers and sizes of the colonies in a concentration-dependent manner (Figure 1(b) and S1(a)). To further investigate the underlying effects, apoptosis detection assay was performed. Flow cytometry analysis demonstrated that liensinine could induce more apoptotic and dead cells (Figure 1(c) and S1(b)). Western blot experiments were also performed to detect several important apoptosis-related proteins after liensinine treatment in A549 and SPC-A1 cell lines (Figures.  S2(a)–S2(b)). Liensinine could induce the upregulation of cleaved-caspase 9, BAX, and cytochrome C, three important signals of mitochondrial apoptosis pathway. Besides, the expression of cleaved-caspase 9 and cleaved-PARP in the downstream of the pathway was also increased. For further confirmation, the expression of cleaved-caspase 3 was detected by IHC experiment (Figure.  S3(a)), which also confirmed that liensinine can induce more apoptosis *in vivo*. Next, we used Z-VAD, a pan-caspase inhibitor to pretreat the NSCLC cells, and flow cytometry analysis demonstrated that Z-VAD significantly decreased the proportion of apoptotic and dead cells after liensinine treatment (Figure 1(d) and S1(c)). Taken together, these results indicated that liensinine exhibits anti-tumor effects *in vitro*.

### 3.2. Liensinine Inhibits Tumor Growth *In Vivo*

**Figure 2:**
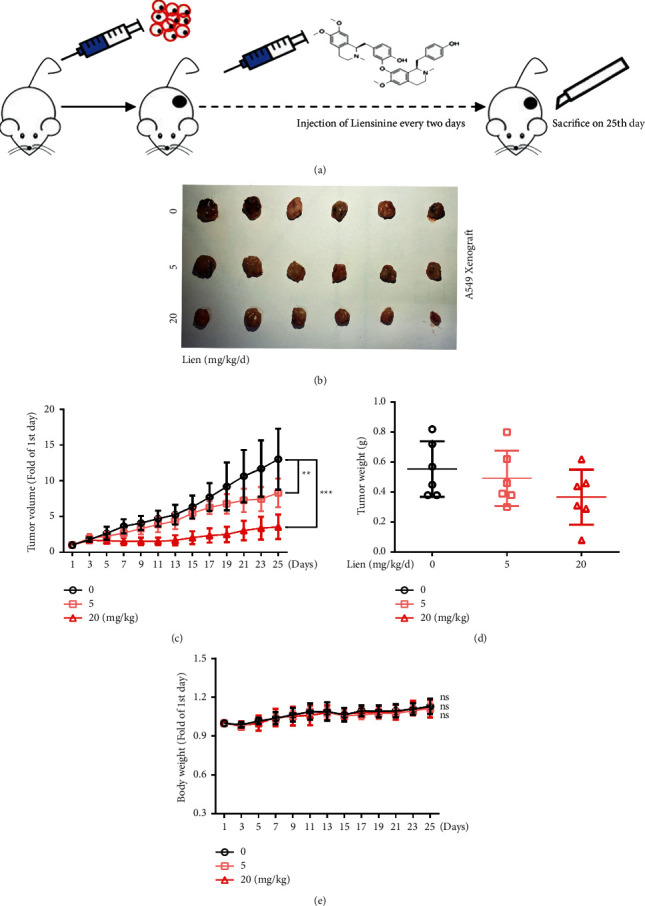
Liensinine inhibits NSCLC proliferation in vivo. (a) A brief flowchart of the experiment design in vivo. A549 xenograft tumor was established and divided into the following groups (control, 5 mg/kg/d, 20 mg/kg/d) and received 25 days of treatment. (b) The image of tumors from different groups. (c) The curves of tumor growth. (d) The weight of tumors. (e) The body weight of mice. The results were shown as means ± SD, ^*∗∗*^*P* < 0.01, ^*∗∗∗*^*P* < 0.001.

Furthermore, murine xenograft tumor model was constructed to verify its anti-tumor effects *in vivo* (Figure 2(a)). A clear reduction trend was observed in both tumor volume and weight after liensinine treatment (Figure 2(b)–2(d)). These results demonstrated that liensinine could inhibit NSCLC tumor growth *in vivo*. Naturally, the side effects and toxicity of liensinine were assessed in tumor-bearing murine. We found no significant difference in the weight of the murine after liensinine treatment compared to the control group (Figure 2(e)). H&E staining was performed on the main organs of mice, and results demonstrated that nothing changed with morphology and histology in the main organs of murine after liensinine treatment compared to the control group (Figure S3(b)). These results implied that liensinine might exert significant anti-tumor effects on tumor tissue, but has no obvious toxicity on normal organs.

### 3.3. Liensinine Promotes Mitochondrial Dysfunction

**Figure 3:**
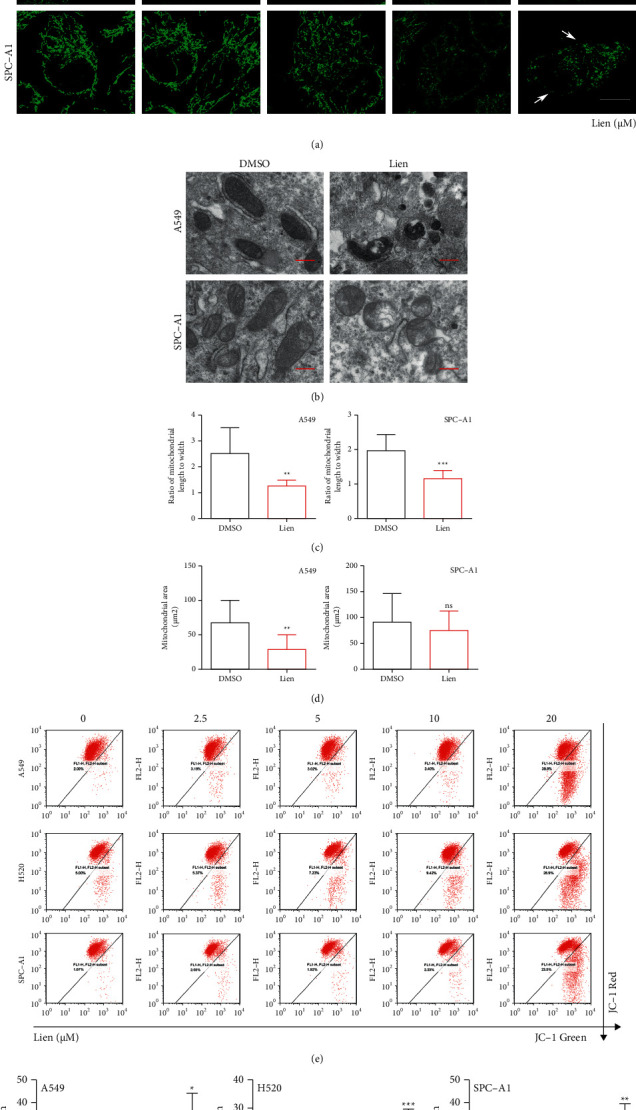
Liensinine promotes mitochondrial dysfunction. (a) A549, H520, and SPC-A1 cells were treated with the indicated concentrations of liensinine for 48h and stained with COX IV antibody. Scale bars: 10 *μ*m. (b–d) A549 and SPC-A1 cells were treated with liensinine or DMSO, and the mitochondria structure was observed by transmission electron microscope. The shape and area of mitochondria were measured and calculated. Scale bars: 0.4 *μ*m. (e–f) A549, H520, and SPC-A1 cells were treated with the indicated concentrations of liensinine for 48h and then detected using JC-1 flow cytometry. Three independent experiments were performed. The results were shown as means ± SD, ^*∗*^*P* < 0.05, ^*∗∗*^*P* < 0.01, ^*∗∗∗*^*P* < 0.001.

Mitochondria are vital and ubiquitous organelles in eukaryotic cells, usually defined as energy factories for survival. Many studies have shown that mitochondria played an undeniable role in the process, whether to promote the growth of tumor cells or enhance the ability of metastasis [17, 23]. In order to explore the underlying mechanism of liensinine in NSCLC cells, we observed the effects of liensinine on mitochondria. We first observed the morphology of mitochondria by staining COX IV, a signature mitochondria-related protein which located in the inner membrane. Liensinine induced more spotty fluorescence but less linear mitochondrial fluorescence signals (Figure 3(a)). In addition, we used transmission electron microscope to observe morphology of cells after liensinine treatment. The morphology of mitochondria became irregular, even transformed from oval to round, and the matrices of the mitochondrial became unclear (Figure 3(b)–3(d). These results demonstrated that liensinine destroyed normal mitochondrial morphology and might intervene the balance between mitochondrial fission and fusion. It has been reported that loss of the mitochondrial inner membrane transmembrane potential could disturb the balance between fission and fusion, thus leading a loss of functions of mitochondria which could maintain normal bioenergy homeostasis [24]. We next performed a flow cytometry assays to detect JC-1 probe. Liensinine caused a significantly decreased red fluorescent intensity compared to green fluorescent intensity, indicating a collapse in mitochondrial membrane potential induced by liensinine (Figures 3(e)–3(f)). Taken together, these observations support that liensinine promotes mitochondrial fission and damage.

### 3.4. Liensinine Induces Autophagosome Accumulation

**Figure 4:**
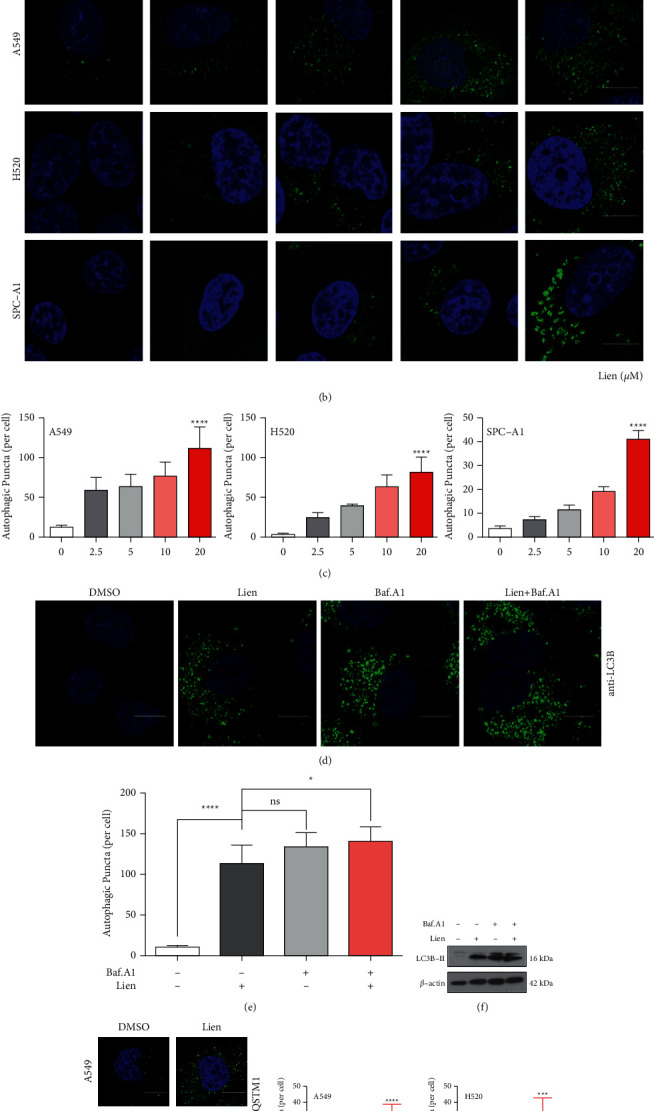
Liensinine induces autophagosome accumulation. (a) A549, H520, and SPC-A1 cells were treated with the indicated concentrations of liensinine for 48h, and Western blot assays demonstrated the LC3B expression. (b–c) LC3B puncta were observed by confocal laser microscopy after staining with LC3B antibody, and autophagic vacuoles were calculated. Scale bars: 10 *μ*m. (d–e) A549 cells were treated with liensinine with or without Baf.A1 and then detected the LC3B puncta by immunofluorescence. LC3B puncta number per cell was quantified. (f) A549 cells were treated with liensinine with or without Baf.A1, and then Western blot assays demonstrated the LC3B expression. (g–h) A549 and H520 cells were treated with liensinine as indicated concentrations and then stained with SQSTM1 antibody. SQSTM1 puncta number per cell was quantified. Scale bars: 10 *μ*m. Three independent experiments were performed. The results were shown as means ± SD, ^*∗*^*P* < 0.05, ^*∗∗∗∗*^*P* < 0.0001.

Autophagy is a self-defense mechanism inherent in mammalian cells, including a process of generated double-membrane structure to engulf damaged organelles by stimulating internally or externally, which could be degraded in lysosomes for energy reuse to cell survival [25]. Therefore, we studied the effects of liensinine on autophagy. We first detected the expression of the autophagosome formation marker LC3B-II by Western blot assays. Liensinine increased the expression of LC3B-II in a concentration-dependent manner compared to control group (Figure 4(a)). The formation of autophagosome was observed by immunofluorescence, and we found that intracellular autophagosome increased after liensinine treatment, as demonstrated by an accumulation of LC3B-positive spot-like structures (Figure 4(b) and 4(c)). Therefore, our results suggested liensinine-induced autophagosome accumulation in NSCLC cells. To identify the possible explanations of the autophagy level induced by liensinine, we incubated the cells with liensinine and Baf.A1. Baf.A1 elevated LC3B-II expression arrived at a similar level in cells with or without presence of liensinine (Figure 4(f)), and the formation of autophagosome detected by immunofluorescence also confirmed that (Figure 4(d)–4(e). These results indicated that liensinine might not involve into the process of the excessive degradation of autophagic flux.

### 3.5. Liensinine Blocks Autophagic Flux

**Figure 5:**
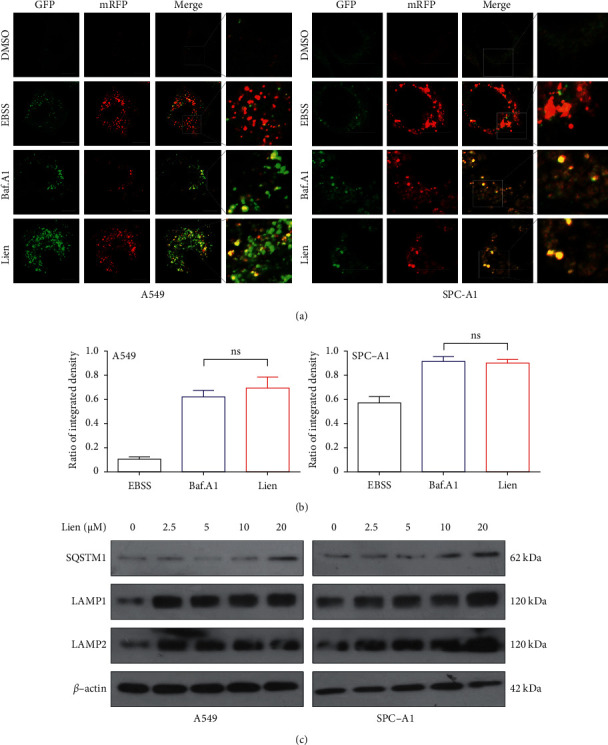
Liensinine blocks autophagic flux. (a–b) A549 and SPC-A1 cells transfected with mRFP-GFP-LC3 virus were treated with EBSS, Baf.A1(100 nm), or liensinine (20 *μ*M), and observed by confocal microscopy. The percentage of GFP in mRFP signals was calculated. Scale bars: 10 *μ*m. (c) A549 and SPC-A1 cells were treated with liensinine as indicated concentrations, and Western blot assays demonstrated the SQSTM1, LAMP1, and LAMP2 expression. Three independent experiments were performed. The results were shown as means ± SD, ns = no significant.

To evaluate the effect of liensinine on autophagic flux, the intracellular SQSTM1, an autophagic flux marker protein which could integrate with autophagosome and be degraded by normal lysosomes, was firstly detected. The immunostaining confirmed that liensinine increased the expression of SQSTM1 and promoted its assembling into aggregates (Figures 4(g) and 4(h)). Moreover, liensinine treatment failed to degrade the SQSTM1 protein compared to the control group in A549 and SPC-A1 cell lines (Figure 5(c)). To further confirm this phenomenon, we used mRFP-GFP-LC3 adenovirus to transfect the NSCLC cells. Under normal condition, this virus labels autophagosome yellow due to merged GFP with mRFP signal, but labels autolysosomes red because GFP signals quench in the abnormal lysosome. Most autolysosomes displayed red because GFP signals quench in EBSS-treated group. However, in liensinine-treated group, like Baf.A1-treated group, the quenching of GFP signal was significantly reduced (Figure 5(a)–5(b)), indicating that the autolysosomes were altered and its normal flux was blocked. To address this, several lysosomal-related proteins were detected including LAMP1 and LAMP2, and the expression was increased by liensinine in a concentration-dependent manner (Figure 5(c)). In addition, we stained A549 and SPC-A1 cells with LTR dye, which acts as a lysosomal acidic indicator. This displayed an increase in the LTR signal in response to liensinine treatment (Figure S4). Taken together, our results proved that liensinine-blocked autophagic flux was independent of reducing the lysosome-associated membrane proteins and does not affect the lysosomal pH.

### 3.6. Liensinine Reduced Mitochondrial Bioenergetic Activity

**Figure 6:**
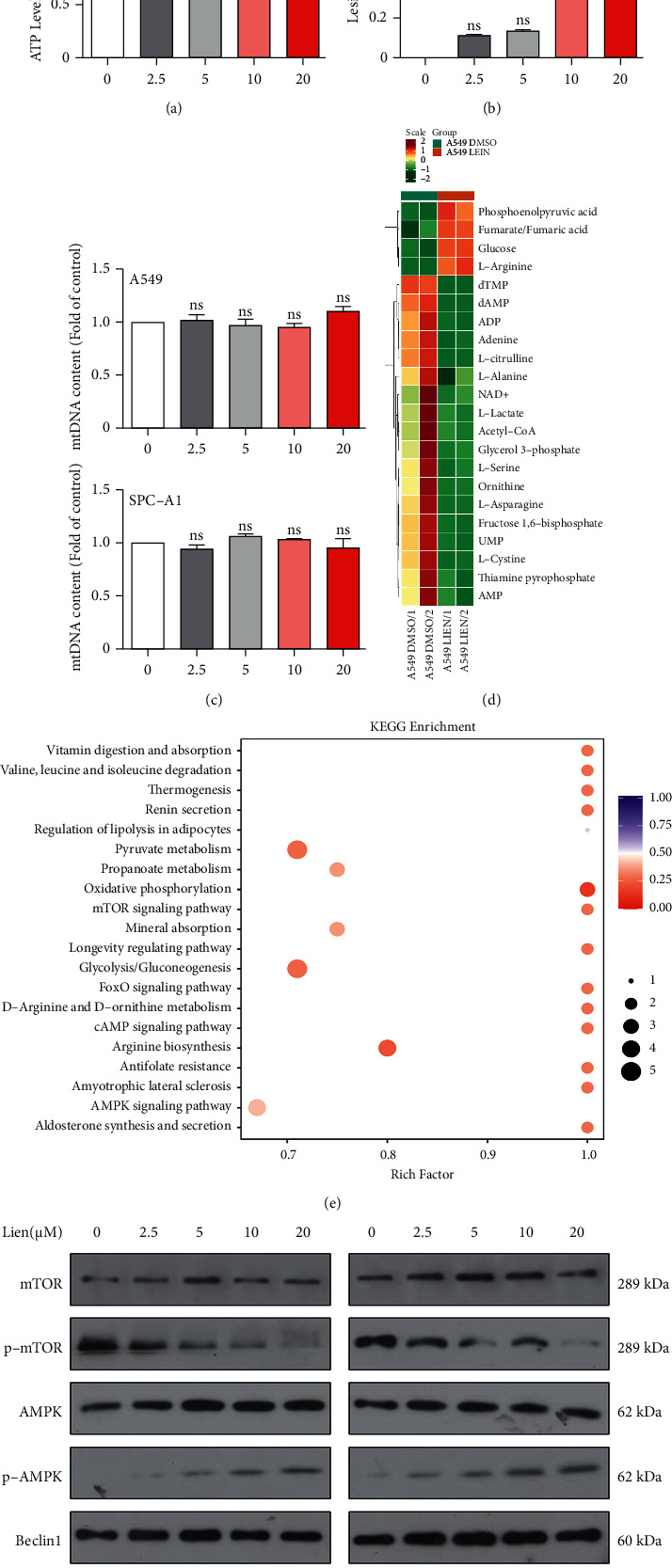
Liensinine reduces mitochondrial bioenergetic activity. (a) A549 and SPC-A1 cells were treated with liensinine as indicated concentrations, and then ATP assay kit was used to evaluate the ATP level. (b-c) A549 and SPC-A1 cells were treated with liensinine as indicated concentrations, and the genomic DNA was extracted and performed LR-PCR and q-PCR to evaluate the mitochondria lesions. Details were shown in methods. (d) A heat map was generated after analysis. The relative expression of metabolites with significant differences was shown in red (upregulation) versus green (downregulation). (e) KEGG pathway analysis for the differential metabolites. (f) A549 and SPC-A1 cells were treated with liensinine as indicated concentrations, and Western blot assays demonstrated the mTOR, p-mTOR, AMPK, p-AMPK, Beclin1, and ULK1 expression. The results were shown as means ± SD, ^*∗*^*P* < 0.05, ^*∗∗*^*P* < 0.01, ^*∗∗∗*^*P* < 0.001, ^*∗∗∗∗*^*P* < 0.0001.

It has been reported that the ATP-dependent proton pump maintains normal functions of lysosomal hydrolase activity [29]. We hypothesized that the dysfunction of autolysosome might be related to the energy changes induced by liensinine. Recently, some study demonstrated that the properly functioning mitochondria in tumor cells are crucial for ensuring their energy supply in contrast to the Warburg effect [30]. We detected the level of ATP, a direct source of energy for the survival of cells, and its level was reduced under liensinine treatment compared to the control group (Figure 6(a)). It seemed that the biogenesis of mitochondria might be impaired in liensinine-treated NSCLC cells. To further confirm this hypothesis, genomic DNA was extracted and performed a long-range PCR assay for detecting mtDNA lesions to compare mitochondrial biogenesis under liensinine treatment. As shown in Figures 6(b) and 6(c), there was an increase in mtDNA lesions but no changes occurred in mtDNA content. It is suggested that mitochondrial damage caused by liensinine might relate to change in cellular metabolism. A targeted metabolism was performed by ultra-performance liquid chromatography to characterize the main metabolic profile of liensinine-treated group compared with control group in A549 cell line. The data presented in Figure 6(d) indicate that there were a total of 22 metabolites with significant changes, among which 18 metabolites were downregulated. After a brief and detailed bioinformatics analysis, decreased levels of glycolysis and tricarboxylic acid (TCA) cycle activity were presented in this system, demonstrating a reduced activity of energy supplying pathways under liensinine treatment in NSCLC cells. AMPK/mTOR pathway is a vital signaling pathway in tumor development, which is not only related to cell energy metabolism [31], but also closely related to autophagy in cells [32]. More interestingly, this pathway was also enriched in our bioinformatics analysis (Figure 6(e)). Therefore, we verified the expression of proteins related to this pathway. As shown in Figure 6(f), the expression of p-AMPK and ULK protein was increased obviously, but the phosphorylation expression of mTOR protein was decreased, which further confirmed liensinine could induce autophagosome accumulation and suggested that liensinine might decrease the bioenergetic activity of NSCLC cells via impacting the AMPK/mTOR pathway.

## 4. Discussion

In the present study, we demonstrated that liensinine inhibited the proliferation of NSCLC cells by inducing apoptosis. More importantly, we observed that liensinine functioned as an autophagy inhibitor via blocking the autophagic flux at the late stage, which significantly accelerated the death of cancer cells. This is possibly because liensinine inhibits the normal function of mitochondrial energy supply and impairs the lysosomal function, thereby proposing liensinine as a potential candidate for cancer therapy.

Increasing evidence has indicated that certain traditional Chinese medicines such as curcumin and resveratrol exhibit great potential in the treatment of cancers [33, 34]. Liensinine is an ingredient extracted from the Plumula Nelumbinis that has been safely used in everyday food products and shown to have a noteworthy anti-tumor effect in recent studies^1^. In this study, the anti-tumor effects and underlying mechanisms of liensinine in nonsmall-cell lung cancer cells are studied and explored for the first time.

We performed assays and demonstrated that liensinine could inhibit the proliferation of NSCLC cells. *In vivo* study further confirmed the anti-tumor effects of liensinine, and we observed no change in the body weight with different groups of mice. The H&E staining of major organs was also normal, which indicates toxic, and side effects of liensinine were controllable. Further results showed that liensinine could induce NSCLC cell apoptosis depending on a mitochondrial pathway. PI and annexin staining presented more death and apoptosis cells under a certain concentration liensinine treatment, and Western blot assays detected several mitochondrial apoptosis-related genes. Liensinine might affect the release of cytochrome C from the outer membrane in mitochondria by activating the pro-apoptotic signal protein BAX, coupling with activated caspase 9 to activate downstream caspase 3 and cleavage PARP protein, thus inducing apoptosis. We also used a pan-caspase inhibitor Z-VAD to perform a rescue experiment and confirmed our assumption that inhibiting caspase could inhibit the apoptosis caused by liensinine.

Aligned with findings of other studies [9, 11, 35], we regard mitochondria as the key target for liensinine treatment in NSCLC cells. We found the morphology and activity of the mitochondria are impaired, with the evidence that mitochondrial fission and fusion are imbalanced and membrane potential drops under liensinine treatment compared with the control group. Next, we observe the situation of autophagy in NSCLC cells. Mainstream studies have shown that autophagy—as a protective mechanism inherent in cancer cells—is activated to maintain cell survival under various stresses. We found that liensinine induces the increase of autophagy levels in NSCLC cells and a mass aggregation of autophagosome. The most important study of liensinine was the discovery that it acts as an inhibitor of mitophagy in breast cancer cells [11]. However, we did not observe mitophagosomes with liensinine treatment in NSCLC cells (data not shown). This might be explained by the difference in cell specificity. We used mRFP-GFP-LC3 adenovirus to transfect the cells, and the results confirmed that liensinine blocked the autophagic flux. We found that LAMP1 and LAMP2 were significantly increased and the pH of lysosome was not increased under liensinine treatment. This suggests that that liensinine-blocked autophagic flux was independent of reducing the lysosome-associated membrane proteins and does not affect the lysosomal pH. Thus, the effect of liensinine on energy metabolism in cancer cells was investigated for the first time. Interestingly, we observed lower ATP levels and more mitochondria DNA lesions in NSCLC cells under liensinine treatment. A targeted metabolic analysis also illustrated that metabolites were generally reduced and mitochondrial bioenergetic activity affected. AMPK/mTOR signaling pathway is highly related to cellular energy in cancer cells [36] and enriched in our KEGG pathway analysis. Western blot experiments verified that liensinine indeed upregulates the level of phosphorylated AMPK and downregulates the level of phosphorylated mTOR, indicating that AMPK/mTOR pathway may be involved in the function of liensinine in NSCLC. Therefore, we hypothesize that liensinine may affect the energy supply, thus causing a dysfunction of lysosome, such as the fusion of lysosome with autophagosome. And it remains to be evaluated with deep work and will also be the focus in our future work.

In recent years, dysregulation of mitochondria DNA and mitochondrial-related metabolism has been related to various aging, cancer, and inflammation-related diseases [21]. Therefore, the exploration of mitochondria-targeted agents is meaningful and necessary for cancer therapy [37]. In our study, liensinine not only presented a significant anti-tumor effect, but also affected metabolism in NSCLC cells. This suggested that liensinine may be involved in the combined treatment of cancers in the future, which of course requires more research.

## Data Availability

The data used to support the findings of the study can be obtained from the corresponding author upon request.

## Supplementary Materials

Supplementary Materials Supplementary Materials. Figure S1. (a) The colony formation of cells was calculated and statistically analyzed. (b) The apoptotic and death cells were calculated and statistically analyzed. (c) The apoptotic and death cells were calculated and statistically analyzed. The results were shown as means ± SD, ^*∗*^*P* < 0.05, ^*∗∗*^*P* < 0.01, ^*∗∗∗*^*P* < 0.001, and ^*∗∗∗∗*^*P* < 0.0001. Figure S2. (a) A549 and SPC-A1 cells were treated with indicated concentrations of liensinine for 48h, and Western blot assays demonstrated the cleaved-PARP, caspase 3, cleaved-caspase 3, cleaved-caspase 9, BAX, and cytochrome *c* expression. (b) The expression of above proteins was statistically analyzed. The results were shown as means ± SD, ^*∗*^*P* < 0.05, ^*∗∗*^*P* < 0.01, ^*∗∗∗*^*P* < 0.001, and ^*∗∗∗∗*^*P* < 0.0001. Figure S3. (a) After the mice were sacrificed, tumors were performed H&E staining and IHC staining using antibody cleaved-caspase 3. (b) The H&E staining of major organs. Scale bars: 50 *μ*m. Figure (s)4. A549 and SPC-A1 cells treated with liensinine were loaded with Lyso-Tracker Red probe and subjected to flow cytometry. Mean fluorescence intensity of LTR was statistically analyzed. Three independent experiments were performed. The results were shown as means ± SD. ^*∗∗∗∗*^*P* < 0.0001.
