# Supplementary material for: Liensinine Inhibits Cell Growth and Blocks Autophagic Flux in Nonsmall-Cell Lung Cancer
Source: J Oncol. 2022 Jul 1;2022:1533779. doi: 10.1155/2022/1533779 (PMC9270144; doi:10.1155/2022/1533779)
Supplement: Supplementary Materials — Figure S1. (a) The colony formation of cells was calculated and statistically analyzed. (b) The apoptotic and death cells were calculated and statistically analyzed. (c) The apoptotic and death cells were calculated and statistically analyzed. The results were shown as means ± SD, ∗P < 0.05, ∗∗P < 0.01, ∗∗∗P < 0.001, and ∗∗∗∗P < 0.0001. Figure S2. (a) A549 and SPC-A1 cells were treated with indicated concentrations of liensinine for 48h, and Western blot assays demonstrated the cleaved-PARP, caspase 3, cleaved-caspase 3, cleaved-caspase 9, BAX, and cytochrome c expression. (b) The expression of above proteins was statistically analyzed. The results were shown as means ± SD, ∗P < 0.05, ∗∗P < 0.01, ∗∗∗P < 0.001, and ∗∗∗∗P < 0.0001. Figure S3. (a) After the mice were sacrificed, tumors were performed H&E staining and IHC staining using antibody cleaved-caspase 3. (b) The H&E staining of major organs. Scale bars: 50 μm. Figure (s)4. A549 and SPC-A1 cells treated with liensinine were loaded with Lyso-Tracker Red probe and subjected to flow cytometry. Mean fluorescence intensity of LTR was statistically analyzed. Three independent experiments were performed. The results were shown as means ± SD. ∗∗∗∗P < 0.0001. [file 1533779.f1.zip › 1533779.f1/Table supplementary.docx]

**Table S1. Primers used for mtDNA integrity analysis.**

|  | **Forward primer** | **Reverse primer** |
| --- | --- | --- |
| 221bp mtDNA | CCCCACAAACCCCATTACTAAACCCA | TTTCATCATGCGGAGATGTTGGA |
| 8.9kb mtDNA | TCTAAGCCTCCTTATTCGAGCCGA | TTTCATCATGCGGAGATGTTGGA |
| mt gene (mtDNA) | CAGGAGTAGGAGAGAGGGAGGTAAG | TACCCATCATAATCGGAGGCTTTGG |
| GAPDH (nucDNA) | CGGAGTCAACGGATTTGGCC | GTGGCAGAGATGGCATGGAC |
